# Circadian Rhythms in Acute Respiratory Distress Syndrome: Molecular Mechanisms and Therapeutic Implications

**DOI:** 10.3390/ijms27104206

**Published:** 2026-05-09

**Authors:** Bao-Tong Liu, Yu Chen, Ya-Lin Zhu, Shi-Chun Ren, Jia-Feng Wang

**Affiliations:** 1 Faculty of Anesthesiology, Changhai Hospital, Naval Medical University, Shanghai 200433, China; 2 Department of Anesthesiology, Naval Medical Center, Naval Medical University, Shanghai 200052, China

**Keywords:** acute respiratory distress syndrome, circadian rhythm, circadian clock proteins, chronotherapy

## Abstract

Acute respiratory distress syndrome (ARDS) is a high-mortality condition lacking targeted treatments. Emerging evidence indicates that circadian rhythm disruption is a key factor in the development of ARDS. Core clock proteins control essential processes, including alveolar–capillary barrier function, inflammation, and tissue repair. The intensive care unit (ICU) environment and underlying illness create double hits that impair biological clocks, leading to a cycle of excessive inflammation and organ damage. This review highlights the central role of circadian rhythms in ARDS. Despite strong preclinical evidence, there are still many challenges for clinical application, including a lack of high-quality human studies and uncertainty about the optimal timing of interventions. Incorporating biological rhythm stabilization into multimodal ARDS management is a prerequisite step toward precision medicine. Future research should focus on mechanistic and translational studies to confirm the safety and effectiveness of chronomedicine in improving long-term patient outcomes.

## 1. Introduction

ARDS is one of the most challenging critical illnesses in the ICU, characterized by persistently high levels of morbidity and mortality. Although substantial progress has been made in mechanical ventilation strategies and supportive care, effective targeted treatments for the core pathological processes—alveolar–capillary barrier disruption and uncontrolled inflammatory responses—are still lacking. Recently, the role of circadian rhythm regulation in ARDS development has gained increasing attention, offering a new perspective for overcoming current therapeutic limitations. This section begins with the clinical challenges of ARDS and then provides a systematic overview of the structure and function of the circadian rhythm system, its natural connection with the respiratory system, and the “double hit” mechanism caused by the ICU environment and the disease itself, thereby establishing a foundation for further exploration of the importance of rhythm regulation and its potential as a therapy.

### 1.1. Method

This narrative review was conducted with a critical synthesis approach. PubMed/MEDLINE and Web of Science were searched using: (“circadian rhythm” OR “biological clock” OR “clock genes” OR “BMAL1” OR “CLOCK” OR “REV-ERBα” OR “PER” OR “CRY”) AND (“acute respiratory distress syndrome” OR “ARDS” OR “acute lung injury” OR “ALI” OR “ventilator-induced lung injury” OR “sepsis” OR “pneumonia”). Articles in English without date restrictions were considered. Additional studies were identified through manual screening of reference lists.

We included peer-reviewed original research and reviews on circadian mechanisms in lung injury or relevant preclinical models, and clinical studies of circadian interventions in critical illness. The search was not exhaustive; rather, it aimed to select the most relevant and representative sources to support a critical narrative synthesis.

### 1.2. Clinical Challenges and Therapeutic Dilemmas in ARDS

ARDS is an acute, widespread inflammatory lung injury characterized clinically by non-cardiogenic pulmonary edema, hypoxemia, and the need for mechanical ventilation support [1]. Common triggers include pneumonia, sepsis, aspiration, and severe trauma. ARDS is a leading cause of respiratory failure in the ICU. A global multicenter study shows that the prevalence of ARDS among ICU patients is about 10%, accounting for 23.4% of those on mechanical ventilation, with mortality rates as high as 30% to 40% [2]. The core pathophysiological process of ARDS involves disruption of the alveolar–capillary barrier, leading to pulmonary edema. Mechanisms such as alveolar epithelial and endothelial injury, inflammation and immune dysregulation, coagulation issues, and ventilator-induced lung injury (VILI) also play critical roles in the progression of ARDS. Epithelial injury plays a key role in determining disease severity [3]. Despite increasing understanding of the underlying mechanisms of ARDS, clinical management remains mainly supportive care, with limited targeted therapies for alveolar–capillary barrier damage and uncontrolled inflammatory responses.

### 1.3. Basic Composition and Function of the Circadian Rhythm System

**Figure 1:**
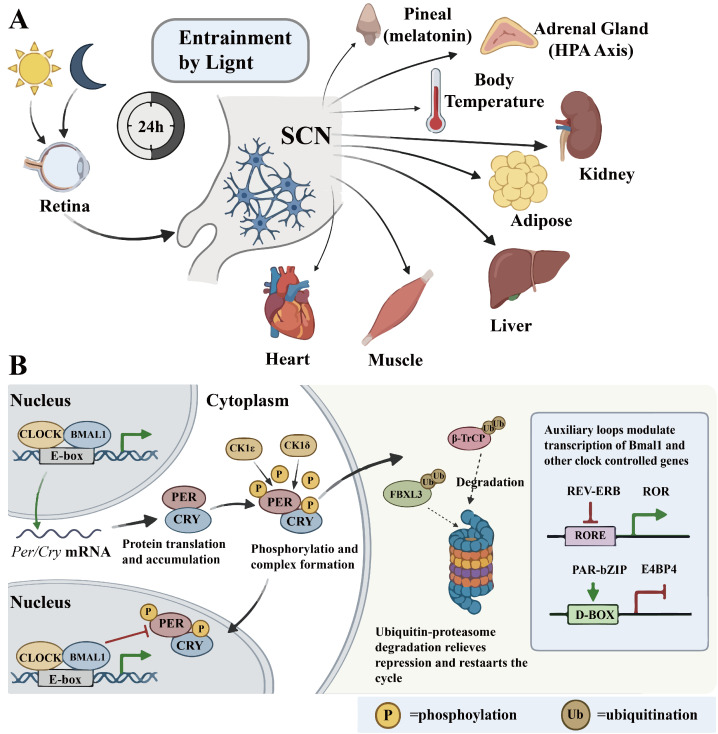
Basic composition and function of the circadian rhythm system (**A**): Central mechanism of circadian rhythm generation. Light entrainment of the SCN master clock and its synchronization of peripheral tissue rhythms via neural, hormonal, and thermal outputs. (**B**): Mechanism of rhythm oscillation generation at the cellular Level. The core CLOCK-BMAL1/PER-CRY transcription-translation feedback loop and its modulation by auxiliary REV-ERB/ROR and PAR-bZIP/E4BP4 loops, with post-translational regulation by CK1δ/ε phosphorylation and β-TrCP/FBXL3-mediated ubiquitin-proteasome degradation. Green arrows, activation; red bars, repression. Green arrows represent stimulatory or promoting effects. Red blunt arrows represent inhibitory or suppressive effects. Other arrows represent directional flow or interaction.

Circadian rhythms are internal timing systems that induce roughly 24-h cycles of physiological and behavioral changes, aligning body functions with the external light–dark cycle to optimize energy utilization and boost survival and reproduction. This mechanism is highly conserved across species from bacteria to humans [4]. In mammals, the suprachiasmatic nucleus (SCN) functions as the principal endogenous oscillator in mammals, with its network of neurons producing highly stable and sustained autonomous rhythms. It detects light signals for synchronization and coordinates the rhythms of peripheral tissues via neural and hormonal signals, achieving overall systemic timing [5]. At the cellular level, these rhythms are driven by a central transcription-translation feedback loop. The CLOCK-BMAL1 heterodimer binds to E-box elements during the day, activating the transcription of *Per* and *Cry* genes. The PER and CRY proteins then build up in the cytoplasm, form dimers, and, after interacting with kinases like CK1δ/ε, move into the nucleus to suppress CLOCK-BMAL1 activity, creating a negative feedback loop. Later, PER and CRY are degraded by ubiquitin pathways involving E3 ligases such as β-transducin repeat-containing protein (β-TrCP) and F-box and leucine-rich repeat protein 3 (FBXL3), which lifts the inhibition and allows CLOCK-BMAL1 activity to restart, beginning a new cycle. This core loop is reinforced and extended by two auxiliary loops: REV-ERB and ROR nuclear receptors bind to RORE elements to regulate *Bmal1* transcription inversely, while proline and acidic amino acid-rich basic leucine zipper (PAR-bZIP) proteins and E4 promoter-binding protein 4 (E4BP4) compete for D-box elements. These interconnected loops promote rhythmic expression of many clock-controlled genes by binding to cis-elements like E-box, RORE, and D-box in gene promoters and enhancers, thus enabling extensive regulation of physiological processes [6] (Figure 1).

### 1.4. Intrinsic Connection Between Circadian Rhythms and the Respiratory System

Circadian rhythms are vital endogenous regulators of respiratory function, causing periodic changes in respiratory center excitability, airway tone, ventilatory efficiency, and pulmonary immune-inflammatory responses via clock genes. The internal circadian clock within airway epithelial cells controls the expression and daily variation in key molecules such as REV-ERBα/β and CXCL5, which influence inflammatory cell recruitment after lipopolysaccharide (LPS) exposure and help reduce pulmonary inflammation [7]. Additionally, circadian rhythms may indirectly affect airway tone and breathing regulation through the autonomic nervous system. Disrupting these rhythms can worsen fluctuations in airway resistance and impair nighttime ventilatory stability, thus worsening respiratory dysfunction [8].

### 1.5. The Double Hit of the ICU Environment and the Disease

**Figure 2:**
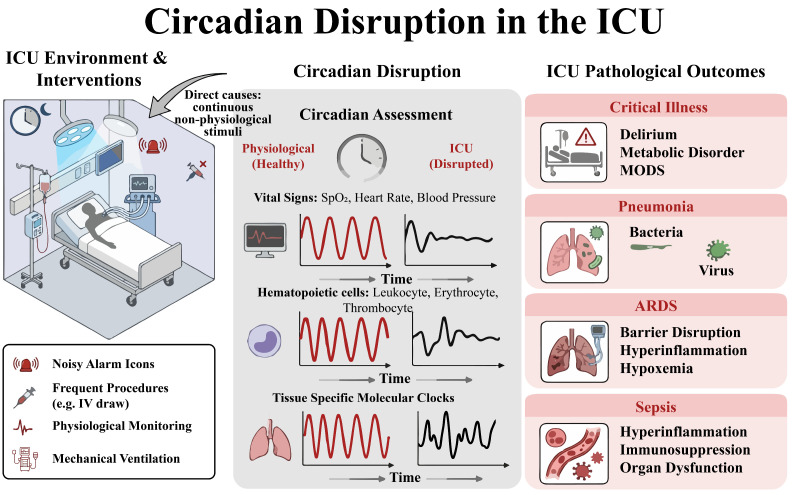
The dual impact of the intensive care environment on disease progression.

Profound hypoxemia and respiratory failure in ARDS patients dictate the need for intensive care, where mechanical ventilation and multisystem support are provided. The ICU environment itself, characterized by continuous artificial lighting and frequent medical interventions, acts as a strong external desynchronizing factor [9]. At the same time, the core pathological drivers, such as uncontrolled pulmonary inflammation, sepsis, and hypoxia, can internally suppress the expression and rhythmicity of core circadian clock genes and disturb the normal cellular molecular clock feedback loop through inflammatory mediators [10,11]. As a result, patients show typical circadian desynchronization, marked by a loss of melatonin secretion rhythm and diminished or disrupted circadian oscillations in physiological parameters like body temperature and heart rate [12]. This disruption of rhythm creates a vicious cycle with the disease process, thereby impairing the body’s ability to regulate inflammation rhythmically, worsening organ damage, and hindering recovery by affecting immune function, metabolism, and muscle homeostasis. This chain of effects increases the risk of multiple organ failure and death [9] (Figure 2).

Current ARDS management continues to focus on organ support, primarily utilizing low tidal volume mechanical ventilation and prone positioning to maintain vital functions. Effective strategies for repairing the alveolar–capillary barrier and modulating excessive immune responses are still missing. In this context, circadian rhythm disruption is not just a consequence of critical illness but a central pathophysiological factor driving disease progression and affecting prognosis. The ICU environment, combined with the disease itself, creates a double hit on the body’s biological clock, causing widespread disruption of physiological rhythms and secondary dysregulation of inflammatory responses. This can worsen pulmonary endothelial and epithelial injury, promote pulmonary edema formation, and potentially increase iatrogenic harm such as VILI. Poor circadian rhythmicity has been identified as an independent risk factor for higher hospitalization risk, longer hospital stays, and increased in-hospital and post-discharge mortality in patients with lower respiratory tract infections.

## 2. Core Mechanisms of Circadian Rhythm Regulation in ARDS

### 2.1. Regulation of Pulmonary Physiological Function by Circadian Rhythms

Circadian variations in respiratory function are observable in mammals, including both experimental animals and humans. Mortola and colleagues demonstrated that ventilatory parameters in rats, such as tidal volume, respiratory rate, and minute ventilation, exhibit intrinsic circadian rhythms independent of locomotor activity [13]. A study controlling for sleep and behavioral influences revealed that basal ventilation in humans remains relatively stable during sustained wakefulness, with minimal fluctuations in end-tidal partial pressure of carbon dioxide (PCO_2_), suggesting that circadian variation in ventilation is primarily mediated by changes in arousal state under normal conditions [14,15]. However, in patients with persistent asthma receiving stable inhaled corticosteroid therapy, forced expiratory volume in one second (FEV1) reaches a low point in the early morning (approximately 04:10) and peaks in the afternoon (around 13:30), with a mean fluctuation of about 170 mL. No covariates, including age, sex, or baseline FEV1, significantly affect the amplitude or phase of this rhythm [16]. This indicates that in disease states, the influence of the body’s internal clock on disease processes becomes more pronounced. At the same time, normal compensatory mechanisms may be partly compromised, highlighting the dual role of the circadian system in both maintaining health and contributing to disease development. Additionally, a study of shift-working police officers showed that long-term circadian disruption was associated with decreased forced vital capacity (FVC) and FEV1, along with higher levels of exhaled nitric oxide and interleukin-2 (IL-2), suggesting impaired lung function and ongoing inflammation [17]. This implies that circadian disruption may weaken pulmonary and systemic immune functions.

### 2.2. Regulation of Pulmonary Cell Function by Circadian Rhythms

The integrity of the alveolar–capillary barrier determines gas exchange efficiency and lung function. It ensures efficient gas exchange, prevents fluid and protein leakage from blood vessels into the alveolar space, and stops uncontrolled entry of inflammatory cells and mediators. A healthy alveolar epithelium is vital for defending against inhaled pathogens and particles. Circadian rhythms act as key regulators of epithelial cell responses to environmental challenges, support immune balance, and facilitate repair. The circadian clock gene *Bmal1* in airway epithelium precisely manages daily changes in neutrophil infiltration by rhythmically controlling the chemokine CXCL5 [18]. Disruption of circadian rhythms can impair lung barrier function, increase vascular permeability, and worsen LPS-induced lung inflammation. Such disruption can alter the actin cytoskeleton and Rap1 signaling pathways by downregulating myosin light chain kinase 4 (Mylk4) and multiple integrins, weakening the integrity of pulmonary endothelial and epithelial layers and making the pulmonary vascular barrier less stable. When additional inflammatory triggers like LPS occur, this existing barrier weakness combines with a heightened immune response, leading to more severe acute lung injury (ALI) [19]. Long-term circadian disruption may also hinder lung tissue repair and heighten the risk and severity of ALI. It can increase the levels of the core clock protein differentiated embryo chondrocyte-expressed gene 1 (DEC1), disrupting its normal rhythmic interaction with p21, leading to sustained p21 levels that cause alveolar type II (AT2) epithelial cells to become senescent and to secrete profibrotic factors, ultimately worsening pulmonary fibrosis [20]. Moreover, circadian rhythms help coordinate the body’s responses to environmental challenges, including pathogens, and influence susceptibility to viral infections. Silencing *Bmal1* in lung epithelial cells or indirectly inhibiting BMAL1 with the REV-ERB agonist SR9009 reduces SARS-CoV-2 entry and replication [21].

Aberrant immune cell activation is a key factor driving lung injury in ARDS. Most immune cells, including monocytes, macrophages, natural killer (NK) cells, mast cells, neutrophils, and lymphocytes, express core clock genes and display roughly 24-h rhythms [22]. Neutrophil levels in the lungs fluctuate with the circadian cycle, enabling the lung to pre-adjust its defensive state at different times to optimize immune response based on varying pathogen exposure risks throughout the activity–rest cycle [18]. By controlling innate immune cell recruitment, especially NK cells, the severity of influenza-induced lung inflammation depends on the time of day, balancing antiviral effectiveness with host tolerance and influencing survival outcomes [11]. Sepsis, a common cause of ARDS, is also affected by circadian mechanisms. BMAL1 deficiency increases programmed death-ligand 1 (PD-L1) expression in macrophages via a signal transducer and activator of transcription 1-dependent (STAT1-dependent) pathway, impairing microbial clearance, promoting T-cell apoptosis, worsening multiorgan failure, and increasing sepsis mortality [23]. The rhythmic changes in pulmonary immune cells coordinate immune defense, energy metabolism, and tissue repair. Disruption or loss of these rhythms, which can occur due to disease, not only results from illness but also actively contributes to immune imbalance, higher infection risk, and resistance to treatment.

### 2.3. Regulatory Mechanisms of Key Circadian Clock Proteins in ARDS

#### 2.3.1. BMAL1

BMAL1 protein, first discovered in 1998, is a bHLH-PAS domain-containing transcription factor that forms a transcriptionally active heterodimer with CLOCK [24]. CLOCK and BMAL1 bind to E-box elements to drive transcription of rhythm-associated factors involved in the circadian negative feedback loop [6]. Multiple studies have thoroughly examined the essential role of BMAL1 in lung injury. and repair.

In ARDS, lung epithelial cells are primary injury targets; their barrier dysfunction leads to pulmonary edema and release of numerous pro-inflammatory mediators that drive neutrophil infiltration and inflammatory cascade [25,26]. Silencing *Bmal1* in lung epithelial cells reduces angiotensin-converting enzyme 2 (ACE2) expression and selectively induces a set of interferon-stimulated genes (ISGs), thereby inhibiting SARS-CoV-2 infection and replication through dual mechanisms involving viral receptor downregulation and enhanced intrinsic immunity [21]. Acute smoke exposure in epithelial cells leads to reduced Sirtuin 1 (SIRT1) deacetylase activity and subsequent acetylation and degradation of BMAL1 protein. Specific knockout of *Bmal1* in mouse lung epithelial cells exacerbates smoke-induced inflammation, and the protective effect of the selective SIRT1 activator SRT1720 is lost in *Bmal1*-deficient mice [27,28]. Glucocorticoid secretion, which is under circadian control, acts as a potent synchronizing signal in many peripheral tissues [29,30,31]. Ray, Loudon, and colleagues demonstrated that rhythmic systemic glucocorticoids exert anti-inflammatory effects via the glucocorticoid receptor (GR) in lung epithelial cells, where GR binds to the CXCL5 promoter, inhibiting transcription and suppressing neutrophil chemotaxis. Using Club cell-specific *Bmal1*-deficient mice, they further showed that *Bmal1* knockout disrupted rhythmic CXCL5 expression, suggesting that BMAL1 is a key node linking the local circadian clock to GR function [18]. However, subsequent studies suggest that this pathway is not the sole mechanism underlying rhythmic neutrophil infiltration [32].

The role of myeloid BMAL1 in ARDS is complex. One study found that macrophage-specific *Bmal1* deletion promotes neutrophil recruitment by upregulating CXCL2 and increasing neutrophil extracellular trap (NET) formation, thereby worsening sepsis-induced lung injury [33]. Conversely, mice with myeloid cell-specific *Bmal1* deficiency showed less circadian variation in VILI and lower mortality at dawn, a time when injury risk is higher [10]. In patients with chronic obstructive pulmonary disease (COPD), some traditional Chinese medicine components increase BMAL1 expression, inhibit p38/JNK MAPK signaling pathway activation, and directly decrease pro-inflammatory cytokine production [34].

BMAL1 functions as a rhythm keeper rather than a simple pro- or anti-inflammatory factor, with effects that depend on cell types, circadian phase, and the nature of the insult. In epithelial cells, BMAL1 maintains barrier integrity and anti-inflammatory signaling [18,27,28], while simultaneously driving ACE2 expression [21], creating a trade-off between homeostasis and viral susceptibility. In myeloid cells, its deficiency exacerbates neutrophil recruitment and NET formation in sepsis-induced lung injury [33] yet reduces mortality in VILI at the circadian nadir [10]. These discrepancies highlight that BMAL1’s net effect depends on the synchronization between circadian amplitude and the timing of environmental challenges. Dissecting these interactions will require cell-type-specific and temporally controlled genetic models.

#### 2.3.2. REV-ERBα

REV-ERBα, first identified in 1989, is an approximately 56 kDa protein encoded on the reverse strand of the ERBA proto-oncogene. It functions as a key transcription factor and repressor within the circadian network and is widely expressed across tissues [35,36]. As a core circadian clock protein and transcriptional repressor, REV-ERBα is involved in various pulmonary pathophysiological pathways, including inflammation, immunity, and metabolism, by regulating downstream gene expression. In aged mice, the time-dependent susceptibility to Streptococcus pneumoniae infection—specifically, increased vulnerability during the rest phase—is lost, a phenomenon attributed to the loss of rhythmic REV-ERBα expression and sustained high baseline levels. This disruption inhibits the downstream Apelin-APJ pathway, impairing alveolar macrophage phagocytic and bactericidal functions [37].

REV-ERBα primarily regulates inflammation via the nuclear factor kappa-light-chain-enhancer of activated B cells (NF-κB) and NOD-, LRR- and pyrin domain-containing protein 3 (NLRP3) inflammasome pathways. In VILI rat models, REV-ERBα expression decreases at both the transcriptional and translational levels. Activation of REV-ERBα with SR9009 significantly reduces pulmonary edema, inflammatory infiltration, and TNF-α production [38]. REV-ERBα inhibits LPS-induced NF-κB activation, which lowers pro-IL-1β synthesis. Since NF-κB activation primes the NLRP3 inflammasome, activating REV-ERBα with GSK4112 downregulates NLRP3 inflammasome components, suppresses caspase-1-mediated pro-IL-1β cleavage, and decreases mature IL-1β release, thereby alleviating LPS-induced ALI [39].

Furthermore, activation of the REV-ERBα signaling pathway directly suppresses inflammatory responses in key lung cell types, including epithelial cells and fibroblasts. Downregulation of pulmonary REV-ERBα expression reduces recruitment of the NCoR1-HDAC3 corepressor complex, relieving transcriptional repression of pro-inflammatory genes and exacerbating inflammation [40]. REV-ERBα may also modulate immune responses through IL-10, as suggested by a mathematical model integrating circadian and inflammatory dynamics [41].

REV-ERBα also affects immune status by influencing macrophage polarization and NET release. LPS stimulation decreases REV-ERBα levels in macrophages, promoting M1 polarization and excessive inflammation [42]; subsequent cell studies showed that SR9009 counters this effect by activating the PI3K-AKT pathway [42]. Normally, REV-ERBα maintains rhythmic expression under physiological conditions. In an LPS-induced mouse ALI model, SerpinB1 knockout eliminated the protective effect of the REV-ERBα agonist melatonin, and melatonin’s ability to inhibit NET formation was lost in SerpinB1-deficient neutrophils and lung tissue. This demonstrates that the REV-ERBα-SerpinB1 axis is a key link between circadian rhythms and neutrophil-driven innate immune lung injury [43].

REV-ERBα’s impact in lung injury depends on rhythmic quality—amplitude and oscillatory robustness—rather than absolute expression levels. The heterogeneity stems from three factors: the nature of circadian disruption, cell-type-specific actions, and the mode of modulation. Rhythmic flattening in aging chronically suppresses phagocytic pathways [37], whereas global suppression in ALI impairs NF-κB/NLRP3 repression [38,39]. Epithelial REV-ERBα regulates barrier function and viral susceptibility [21], while myeloid REV-ERBα controls neutrophil recruitment and macrophage polarization [33,42]. Chronic loss through genetic deletion or aging produces adaptive changes, whereas acute pharmacological activation may bypass feedback mechanisms and enhance corepressor recruitment [38,39]. These converging lines of evidence indicate that targeting REV-ERBα in ARDS requires precise calibration of intervention timing and cell-type specificity, rather than uniform agonism or antagonism.

#### 2.3.3. RORα

RORα is a crucial part of the core circadian molecular network that promotes *Bmal1* transcription by binding to RORE elements, thereby maintaining circadian rhythms in peripheral tissues, including the lung [44]. In primary mouse lung fibroblasts, ethanol decreases the expression of BMAL1 and RORα. The RORα agonist SR1078 counteracts ethanol-induced upregulation of transforming growth factor β (TGFβ) and α-smooth muscle actin (α-SMA), whereas the inverse agonist SR3335 mimics ethanol’s profibrotic effects, suggesting that RORα could be a druggable target for controlling abnormal repair processes in ARDS [45].

RORα evidence in lung injury is currently confined to fibroblast biology. Whether it exhibits cell-type-specific heterogeneity analogous to BMAL1 or REV-ERBα—particularly divergent effects in epithelial versus immune cells—remains unexplored. Mapping its circadian dynamics across pulmonary cell types and comparing rhythmic activation with constitutive agonism are critical next steps for ARDS therapy.

#### 2.3.4. PER2

The PER2 protein, encoded by *Per2*, was initially identified in Drosophila mutagenesis screens [46]. It forms a heterodimer with CRY to provide negative feedback on BMAL1-CLOCK-driven transcription [6]. Normal PER2 function is essential for circadian variation in sepsis severity, which peaks at night, by modulating macrophage responses to toll-like receptor 2 (TLR2) signaling [47].

In the context of HIV, the trans-activator of transcription (TAT) protein upregulates miR-126-3p to inhibit SIRT1, leading to dysregulated acetylation and decreased BMAL1 and PER2 expression, thereby disrupting pulmonary clock homeostasis and promoting IL-6, IL-8, and TNF-α secretion, which drive lung inflammation and injury [28]. In rheumatoid arthritis-associated interstitial lung disease, melatonin upregulates PER2 and CRY2 expression, reducing CCL2, CCL3, and CCL4 secretion, thereby attenuating monocyte-macrophage recruitment and mitigating pulmonary inflammation and fibrosis [48].

PER2 expressed in AT2 cells plays a key role in lung inflammatory defense. Bright light or pharmacological enhancement of PER2 rhythmic amplitude increases BPIFB1 expression, which inhibits NF-κB activation, boosts phagocytic capacity, and reduces epithelial sodium channel expression, collectively improving outcomes in bacterial ALI models [49,50].

Chronic circadian disruption suppresses *Per1* and *Per2* expression in NK cells, decreasing eomesodermin expression, impairing cytotoxicity and cytokine production, and promoting pulmonary inflammation and tumor progression [51].

PER2 acts as a versatile amplitude modulator with mechanistically heterogeneous yet functionally consistent effects across cell types. Its net protective impact depends on cell-type-specific wiring, but sustained suppression produces uniformly deleterious outcomes distinct from physiological circadian fluctuation [28,47,48,51]. This highlights that oscillatory quality, not mere presence or absence, determines functional efficacy. Harnessing PER2’s protective potential requires cell-type-specific models and temporally resolved interventions.

#### 2.3.5. CLOCK

The *Clock* gene, mapped to mouse chromosome 5 in 1994, is a key part of the mammalian circadian oscillator [52]. CLOCK forms a heterodimer with BMAL1 to promote circadian transcription [6]. After cecal ligation and puncture-induced sepsis, *Clock*-deficient mice showed better survival, lower levels of inflammatory cytokines, improved bacterial clearance, and less ALI, suggesting that CLOCK may have pro-inflammatory and pro-injury effects in this context [53].

CLOCK-BMAL1 heterodimer components are functionally divergent: CLOCK is broadly pro-inflammatory, whereas BMAL1’s effects are cell-type- and context-specific. This discrepancy likely reflects CLOCK’s clock-independent proinflammatory activity, directly enhancing NF-κB transcriptional and cytokine expression, rather than circadian disruption per se [54]. NPAS2 may partially compensate for CLOCK by dimerizing with BMAL1 to sustain circadian oscillation [55,56]; however, whether this occurs in relevant pulmonary cell types during sepsis-induced lung injury remains untested. Current evidence is limited to a single model lacking cell-type or temporal resolution. Conditional knockout models and NPAS2 profiling are needed to distinguish rhythmic from non-rhythmic functions of CLOCK in critical illness.

**Figure 3:**
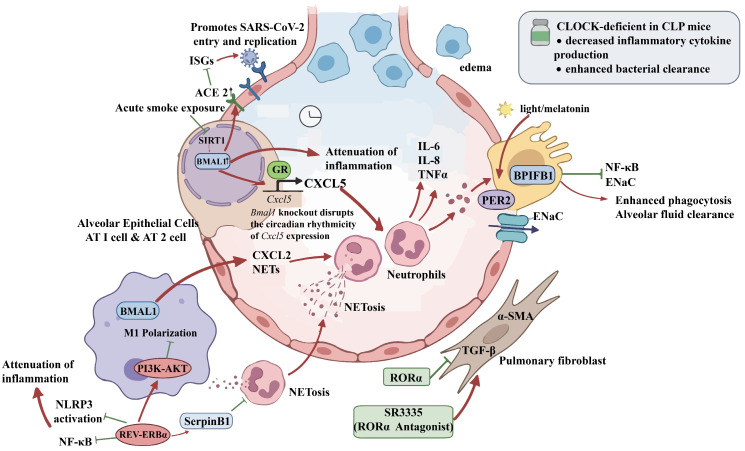
Roles of core circadian proteins in ARDS. Red arrows represent stimulatory or promoting effects. Green blunt arrows represent inhibitory or suppressive effects. Other arrows represent directional flow or interaction.

In summary, core circadian clock proteins may influence the occurrence and progression of ARDS by regulating pulmonary epithelial barrier function, inflammatory responses in immune cells such as macrophage polarization and neutrophil extracellular trap formation and fibroblast-mediated fibrotic process (Figure 3).

## 3. Rhythm-Based Prevention and Treatment Strategies for ARDS

### 3.1. Integrated Chronotherapy

#### 3.1.1. Optimization of Light Environment

Light is the most effective environmental signal for circadian entrainment. Retinal ganglion cells send light signals to the SCN, which then controls pineal melatonin secretion and sleep–wake cycles [57,58]. Light influences a wide range of circadian physiological processes, including sleep–wake patterns, blood pressure, heart rate, respiratory function, hormone production, and immune response [59]. Typical ICU lighting conditions are inadequate for maintaining circadian stability, characterized by dim daytime light and frequent nighttime illumination from medical procedures, which together cause circadian disruption in patients [60,61]. Animal studies have demonstrated that bright light can have protective effects in models of ALI and myocardial ischemia–reperfusion [49,50]. In a pilot clinical trial, postoperative blue light exposure in patients with appendicitis was feasible and reduced serum IL-6 and IL-10 concentrations, suggesting translational relevance [62]. In parallel, a proof-of-concept randomized controlled trial (RCT) indicated that a multicomponent ICU design incorporating dynamic lighting reduced the incidence and severity of delirium while enhancing circadian alignment [63]. However, other research showed that light intervention did not lower overall delirium rates but significantly reduced severe agitation and hallucinations [64,65]. These conflicting findings may result from differences in control group lighting levels, intervention specifics, or the complex nature of circadian disruption in the ICU. Despite these inconsistencies in non-pulmonary endpoints, light therapy remains an adjunctive component of ICU care strategies, meriting further research alongside early mobilization, sleep preservation, and pain control. Yet its translation to ARDS specifically faces substantial gaps: standardized protocols for illuminance, photoperiod, wavelength, and timing relative to disease onset are lacking; the protective effects observed in animal models of ALI and myocardial ischemia–reperfusion await validation in human pulmonary pathology; and the impact of blue light on alveolar epithelial integrity and lung-specific inflammation remains entirely uncharacterized. These limitations reflect both the biological complexity of diurnal-nocturnal species differences and the methodological challenges of implementing dynamic lighting within ICU workflows.

#### 3.1.2. Adjustment of Feeding Rhythms

In addition to light signals, food intake acts as a zeitgeber for peripheral clocks in tissues like the liver, fat, and muscle. Irregular eating patterns, due to shift work, late-night eating, or social jet lag, can cause peripheral clocks to become out of sync with the central SCN clock, leading to metabolic disorders [66,67,68]. Continuous feeding in the ICU can disrupt circadian rhythms and impair glucose tolerance [69], a known risk factor for poor sepsis outcomes [70,71]. However, clinical trials of time-restricted feeding for circadian realignment in ARDS or critically ill patients are lacking.

#### 3.1.3. Chrono-Ventilation Strategy

In the current diagnostic and therapeutic approach for ARDS, treatment is shifting from universal supportive strategies to personalized precision models. Although lung-protective mechanical ventilation provides essential support, the mechanical stress it imposes can cause VILI, and the lung’s susceptibility to such injury varies with the circadian rhythm. Core clock proteins such as BMAL1 and REV-ERBα not only regulate physiological cycles but also directly modulate responses to mechanical stress [10,38]. These findings provide a theoretical basis for a “chronoventilation” method that adaptively adjusts settings such as tidal volume and positive end-expiratory pressure (PEEP) based on the time of day. However, clinical evidence for this approach is currently lacking, and its safety and effectiveness need to be validated.

#### 3.1.4. Chrono-Administration of Medication

The principle of chronotherapy—aligning drug administration with circadian rhythms to optimize efficacy and minimize toxicity—has been most extensively validated in oncology. Chronomodulated administration of 5-fluorouracil (peak delivery at 4:00 am) and oxaliplatin (peak delivery at 4:00 pm) significantly reduced mucositis and diarrhea while improving tumor response rates [72,73]. Similarly, irinotecan and epidermal growth factor receptor (EGFR) inhibitors demonstrated enhanced therapeutic indices when timed according to circadian patterns [74]. Perhaps most relevant to immune-mediated conditions, retrospective analyses revealed that immune checkpoint inhibitors administered in the morning or early afternoon were associated with superior overall survival compared to late afternoon or evening dosing, attributed to circadian regulation of T-cell trafficking and the tumour immune microenvironment [75]. Beyond oncology, time adjustment of medication administration to align with peak target activity has also enhanced therapeutic effectiveness in rheumatoid arthritis. IL-6 secretion in healthy young adults follows a biphasic circadian pattern, with troughs around 08:00 and 21:00 and peaks around 19:00 and 05:00 [68]. Morning symptoms peak alongside nocturnal IL-6 elevation, and evening administration of modified-release prednisone more effectively suppresses this cytokine surge and alleviates morning symptoms compared to morning dosing. Baricitinib, a selective Janus kinase (JAK) inhibitor that blocks IL-6 signaling, has a short half-life, indicating that timed dosing could improve its anti-inflammatory effects, as shown in an animal model of arthritis [71,72]. These circadian-modulated pathways in cancer immunotherapy and rheumatoid inflammation offer a mechanistic rationale for ARDS, where IL-6-driven hyperinflammation is a central pathogenic feature. For patients with the hyperinflammatory subphenotype of ARDS, characterized by significantly elevated inflammatory markers, glucocorticoids and targeted biologics may provide survival benefits. However, the translation of chronotherapy to ARDS and sepsis remains in its infancy. Currently, no prospective trials have specifically evaluated chronomodulated anti-inflammatory agents, corticosteroids, or antimicrobial therapy in ARDS. The field awaits studies that leverage the circadian variation in NF-κB activity, cortisol secretion, and immune cell trafficking to optimize drug delivery windows. Therefore, incorporating chronotherapy into ARDS precision management aligns with circadian pathophysiology in ARDS precision management, supported by robust preclinical evidence from animal and cellular studies demonstrating that circadian disruption amplifies pulmonary inflammation and impairs host defense through clock-gene-dependent mechanisms. However, high-quality clinical studies in ARDS patients are still needed. In summary, although preclinical and early-phase evidence supports the biological plausibility of circadian interventions, clinical validation for ARDS-specific applications remains sparse for light therapy and nonexistent for feeding-based approaches, with chronoventilation confined to experimental models. The convergence of circadian-modulated inflammatory pathways supports integrating biological rhythm restoration into multimodal ICU care. Future research must prioritize translational trials to standardize light protocols, validate ventilator timing parameters, and establish safe dosing windows for chronomodulated therapies.

### 3.2. Clinical Translation of Circadian Small-Molecule Modulators

The treatment of ARDS has shifted from broad supportive care to targeted precision approaches. Among new strategies, small-molecule modulators of key circadian clock proteins hold unique promise to address the full range of ARDS pathology by regulating the circadian system that manages inflammation, metabolism, apoptosis, and repair. Unlike traditional anti-inflammatory drugs, these modulators may not only reduce excessive inflammation but also restore tissue balance by resetting disrupted circadian rhythms.

#### 3.2.1. REV-ERBα Agonist

SR9009, a REV-ERBα agonist, has been shown to reduce VILI in various models by decreasing leukocyte infiltration and inflammatory levels [38]. It influences several pathways involved in VILI, including the NLRP3 inflammasome, NF-κB, and JNK/ERK-3 signaling [37,39,76,77]. In sepsis models, SR9009 lessens cytokine storms by inhibiting TNF-α and IL-6 and modulates the PD-L1 immune checkpoint pathway, leading to improved outcomes [23,62,78].

#### 3.2.2. RORα Agonist

SR1078, an RORα agonist, effectively reverses ethanol-induced pro-fibrotic responses, significantly reducing TGFβ and α-SMA expression [45]. This indicates that RORα agonists could have therapeutic utility in preventing or reducing post-ARDS pulmonary fibrosis by resetting the disrupted circadian clock in lung tissue and inhibiting fibroblast-to-myofibroblast differentiation.

Regretfully, no clinical trials of SR9009 or SR1078 in ARDS or critical illness have been conducted to date.

#### 3.2.3. Melatonin

Melatonin, a hormone secreted rhythmically by the pineal gland, has powerful antioxidant and anti-inflammatory effects through receptor-dependent and independent mechanisms. Exogenous melatonin has shown protective effects in various lung injury models, due to its ability to neutralize free radicals, boost antioxidant enzymes, inhibit NF-κB and NLRP3 inflammasome activation, and provide anti-apoptotic and mitochondrial protective effects [79,80,81]. Recent research has revealed an inherent connection between melatonin and the circadian clock; for example, its cardioprotective effects in ischemia–reperfusion injury are lost in *Per2* knockout models, highlighting PER2 as a key downstream mediator [82].

Clinically, multiple trials have consistently employed nighttime dosing to align with physiological melatonin secretion peaks, demonstrating reduced mortality, thrombosis, sepsis incidence, and organ failure scores in severe COVID-19 and surgical sepsis [83,84,85,86]. The convergence of these nighttime administration studies—spanning oral doses from 10 mg to 200 mg and intravenous regimens of 60 mg/day—suggests that synchronization with endogenous circadian rhythms, rather than absolute dose, underlies therapeutic efficacy, as corroborated by Sánchez-García et al.’s observation that different melatonin doses (50, 100, 200 mg) produced similar clinical outcomes when given at the same nighttime interval [84]. This interpretation is reinforced by contrasting findings from daytime administration. Alamili et al. observed only modest, inconsistent anti-inflammatory effects when melatonin was given during daytime [87].

A shared limitation across the circadian modulator landscape—from preclinical REV-ERBα and RORα agonists to clinically tested melatonin—is the absence of direct circadian biomarker validation. Although melatonin trials are positioned within chronobiological paradigms and employ nighttime dosing consistent with physiological secretion patterns, none measured core body temperature rhythms, cortisol secretion curves, or peripheral clock gene expression to objectively confirm that rhythm restoration is the mechanistic basis of clinical improvement. The chronotherapeutic claim for melatonin thus remains inferential, relying on its established role as a circadian pacemaker rather than empirically demonstrated phase stabilization. Additionally, most trials were single-center, employed varying doses and routes of administration and lacked standardized definitions of “nighttime” dosing across geographic and seasonal variations.

## 4. Challenges and Perspectives

### 4.1. Major Barriers to Clinical Translation

Although extensive preclinical studies have confirmed that circadian rhythm regulation modulates neutrophil infiltration, alveolar inflammation, and vascular permeability in ARDS [59], translating these findings into clinical applications faces several challenges.

#### 4.1.1. Species and Model Limitations

Most existing evidence comes from animal models or cell-based experiments, with a notable lack of high-quality randomized controlled trials involving ARDS patients, making it difficult to properly assess the safety and effectiveness of interventions. Direct extrapolation from these preclinical systems to human ARDS entails substantial biological and methodological risks. First, species-specific circadian architectures fundamentally differ: rodents are nocturnal, with peak activity and feeding during the dark phase, whereas humans are diurnal, concentrating these behaviors in daylight hours [88]. This inversion alters the phase relationship between clock gene expression and immune cell trafficking, complicating the translation of chronotherapeutic windows derived from murine sepsis or lung injury models. Second, while animal experiments employ standardized conditions that enable mechanistic clarity, human ARDS presents a confluence of circadian disruption, polypharmacy, multiorgan dysfunction, and inflammatory heterogeneity that complicates the isolation of chronotherapeutic effects.

#### 4.1.2. ICU Implementation Challenges

The ICU environment is inherently complex, with patients often receiving multiple concurrent treatments such as mechanical ventilation, sedation, and pain management, and ongoing nutritional support [89]. These conventional interventions frequently operate at cross-purposes with chronotherapeutic goals, creating implementation barriers that extend beyond biological efficacy to bedside feasibility. Sedation practices, a cornerstone of ARDS management [90], profoundly suppress endogenous melatonin secretion and obliterate patient-perceived light–dark cues, undermining the very entraining signals that light-based or melatonin-based chronotherapy seeks to leverage [91]. ICU chronointerventions require coordinated protocol adherence across nursing shifts, respiratory therapists, and physician teams—an implementation challenge that extends beyond individual patient physiology to encompass staffing logistics, handoff communication, and institutional culture.

#### 4.1.3. Patient Heterogeneity

ARDS patients show significant clinical variability, including differences in causes, inflammatory subtypes, and gene expression profiles, yet reliable biomarkers to identify patient groups most likely to benefit from rhythm-based interventions are currently missing [3]. This etiological heterogeneity likely extends to circadian biology: the primary insult—whether infectious, chemical, or traumatic—may differentially entrain or disrupt peripheral clocks, generating distinct chronotherapeutic vulnerabilities. In sepsis-related ARDS, the primary driver is pathogen-associated molecular pattern (PAMP)-induced systemic inflammation, where LPS directly suppresses pineal melatonin synthesis via TLR4-NF-κB signaling and disrupts SCN-driven temperature rhythms, producing a state of central and peripheral circadian collapse [92,93]. By contrast, in acid aspiration-induced ARDS, the insult is localized chemical injury to alveolar epithelium with secondary neutrophilic inflammation; the circadian disruption may be predominantly peripheral, driven by local tissue damage and hypoxia rather than systemic endotoxemia [94,95]. In this context, clock gene expression in lung-resident cells may desynchronize from the intact SCN, creating a tissue-specific phase misalignment. These mechanistic distinctions imply that rhythm-based interventions may require etiology-specific tailoring. However, no study has prospectively compared circadian biomarker profiles or chronotherapy responsiveness across ARDS etiologies, leaving this precision approach entirely speculative.

#### 4.1.4. Model Complexity and Validation Needs

Preclinical ARDS models further complicate the extrapolation of rhythm-based findings due to divergent circadian disruption profiles. LPS models produce synchronized inflammatory kinetics suitable for chronotherapy proof-of-concept but lack mechanical ventilation components; VILI models demonstrate phase-dependent injury severity related to ventilation timing; sepsis models exhibit progressive circadian disruption with substantial inter-animal variability. These model-specific differences carry critical implications: LPS models may overestimate treatment efficacy due to temporal predictability; VILI models address mechanical ventilation timing but not infectious etiologies; sepsis models require phase-stratified analysis to disentangle time-dependent effects from disease-stage confounding. No existing model integrates all three dimensions of clinical ARDS—pathogen, mechanical stress, and host response—nor do studies systematically compare circadian biomarkers across models using standardized protocols. This experimental fragmentation parallels clinical heterogeneity and underscores the need for multi-model validation and prospective trials stratified by both etiology and circadian phenotype.

### 4.2. Future Research Directions

#### 4.2.1. Mechanistic Research

Future studies should further clarify the core molecular mechanisms by which circadian rhythms influence ARDS. Specifically, examining the spatiotemporal expression patterns of key clock proteins, such as BMAL1, REV-ERBα, and PER2, across different lung cell types (e.g., epithelial cells, endothelial cells, macrophages), and their roles at various stages of lung injury and repair, is necessary. Additionally, the participation of the intrinsically photosensitive retinal ganglion cell-suprachiasmatic nucleus (ipRGC-SC) pathway in regulation within humans, as seen in animal models, particularly how light signals affect pulmonary inflammation and immune responses through non-image-forming visual pathways [62,96,97], remains to be investigated. Moreover, the mechanisms underlying the interaction between rhythm regulation and current therapies (such as mechanical ventilation, glucocorticoids) remain unclear. They could benefit from systematic analysis using multi-omics approaches and conditional gene-knockout animal models.

#### 4.2.2. Clinical Research

In terms of clinical research, rigorous randomized controlled trials are urgently needed to evaluate the safety and efficacy of chronotherapy strategies in ARDS patients. Several directions merit particular attention: first, standardization of dynamic light intervention protocols, including optimizing key parameters such as light intensity, spectral composition, timing, and duration [98]; second, validation of the feasibility of timed drug administration, especially the optimal dosing windows for medications like glucocorticoids [99] and JAK inhibitors [100]; and third, exploratory studies of the chronoventilation strategy to assess whether dynamic adjustment of mechanical ventilation parameters based on circadian rhythms provides lung-protective effects. Furthermore, functional imaging modalities such as functional magnetic resonance imaging(fMRI) and positron emission tomography(PET), combined with electrophysiological monitoring, could be used to verify the effects of rhythm-based interventions on central and peripheral neural circuit function.

#### 4.2.3. Individualized Strategies

Given the high heterogeneity of ARDS, future rhythm-based interventions should shift toward personalized approaches. Reliable biomarkers—such as peripheral blood clock gene expression profiles, melatonin rhythmicity characteristics, and circadian fluctuation patterns of inflammatory cytokines—should be identified to select patient subgroups likely to benefit from these interventions. Wearable devices and continuous physiological monitoring technologies can enable real-time assessment of patients’ circadian rhythm status, enabling dynamic adjustments to intervention protocols. Moreover, artificial intelligence and machine learning techniques can integrate multidimensional data (clinical, molecular, and physiological) to develop individualized models for rhythm regulation, supporting precision treatment decision-making. Incorporating rhythm-based therapies into multimodal ARDS management can shift the treatment approach from symptomatic relief to root-cause targeting, ultimately enhancing patient outcomes.

## 5. Conclusions

As an endogenous timing system governing physiological homeostasis, circadian rhythm disruption promotes alveolar inflammation and barrier dysfunction in ALI and ARDS. Core clock proteins, including BMAL1, CLOCK, REV-ERBα, and PER2, are widely expressed in key lung cell types, where they regulate barrier function, inflammatory responses, oxidative stress, and tissue repair throughout the disease process. Circadian disruption exacerbates lung injury, amplifies inflammation, and impairs repair, while ARDS reciprocally perturbs circadian rhythms, forming a vicious cycle that elevates the risk of adverse outcomes in critically ill patients. Based on this mechanistic understanding, rhythm-based regulatory strategies have emerged as a mechanistically rational target for preventing and treating ALI/ARDS. These include both chronotherapy, which aligns diagnostic and therapeutic approaches with diurnal physiological changes, and circadian small-molecule interventions that directly restore the disrupted clock network. Together, they represent a shift toward precision medicine and addressing root causes. Although preclinical studies have shown their efficacy, challenges remain regarding tissue targeting, timing of interventions, and clinical implementation. Moving forward, adopting the concept of chronomedicine and prioritizing the stabilization and restoration of biological rhythms as therapeutic targets requires prospective clinical validation. Through interdisciplinary collaboration, further understanding of subtype-specific rhythm disruption mechanisms, rigorous clinical testing of chrono-interventions and rhythm modulators, and incorporating environmental cues such as light, nutrition, and sleep into standard ICU care, a comprehensive multimodal approach can be developed. This strategy offers hope for overcoming current therapeutic challenges in ALI/ARDS and improving long-term patient outcomes.

## Data Availability

No new data were created or analyzed in this study. Data sharing is not applicable to this article.

## Abbreviations

The following abbreviations are used in this manuscript:

ACE2	Angiotensin-Converting Enzyme 2
AKT	Protein Kinase B
ARDS	Acute Respiratory Distress Syndrome
ALI	Acute Lung Injury
AT2	Alveolar type II cells
bHLH-PAS	basic Helix-Loop-Helix/Per-Arnt-Sim
BMAL1	Brain and Muscle ARNT-Like 1
CLOCK	Circadian Locomotor Output Cycles Kaput
CRY	Cryptochrome
FEV1	Forced Expiratory Volume in One Second
FVC	Forced Vital Capacity
GR	glucocorticoid receptor
HDAC3	Histone Deacetylase 3
ICU	Intensive Care Unit
JAK	Janus Kinase
JNK	c-Jun N-terminal Kinase
LPS	Lipopolysaccharide
MAPK	Mitogen-Activated Protein Kinase
NCoR1	Nuclear Receptor Corepressor 1
NF-κB	Nuclear Factor kappa-light-chain-enhancer of activated B cells
NK	Natural Killer Cells
NLRP3	NOD-, LRR- and pyrin domain-containing protein 3
PCO_2_	Partial Pressure of Carbon Dioxide
PD-L1	Programmed Death-Ligand 1
COPD	Chronic Obstructive Pulmonary Disease
PER	Period
PI3K	Phosphoinositide 3-Kinase
REV-ERBα	Reverse ErbA-related protein α
ROR	Retinoid-related Orphan Receptor
SCN	Suprachiasmatic Nucleus
SIRT1	Sirtuin 1
TGFβ	Transforming Growth Factor beta
TLR	Toll-Like Receptor
VILI	Ventilator-Induced Lung Injury
α-SMA	α-Smooth Muscle Actin
